# Association between 24-hour urine volume and 28-day intensive care unit mortality in sepsis patients: a multi-center retrospective cohort study

**DOI:** 10.3389/fmed.2024.1486232

**Published:** 2024-11-26

**Authors:** Yuzhan Lin, Weiguo Lin, Cheng Fu, Ruixue Sun, WeiLi Hong, Xinglin Chen, Shaorong Yan

**Affiliations:** ^1^Department of Clinical Laboratory, The Third Affiliated Hospital of Wenzhou Medical University, Ruian, Zhejiang, China; ^2^Department of Urology, The Third Affiliated Hospital of Wenzhou Medical University, Ruian, Zhejiang, China; ^3^Department of Clinical Laboratory, Ruian Traditional Chinese Medicine Hospital, Ruian, Zhejiang, China; ^4^Department of Emergency Intensive Care Unit, The Third Affiliated Hospital of Wenzhou Medical University, Ruian, Zhejiang, China; ^5^Department of Epidemiology and Biostatistics, Empower U, X&Y Solutions Inc., Boston, MA, United States

**Keywords:** dose-response relationship, intensive care unit, mortality, sepsis, urine volume

## Abstract

**Background:**

Sepsis is defined as a dysregulated host response to infection that results in life-threatening organ dysfunction. The 24-hour urine volume plays a crucial role in assessing the prognosis of septic patients. This study aims to investigate the relationship between 24-hour urine volume and 28-day intensive care unit (ICU) mortality in septic patients and exploring the dose-response relationship between these variables.

**Methods:**

This retrospective cohort study analyzed data from 7,218 sepsis patients in the eICU Collaborative Research Database. Logistic regression models and generalized additive models were used to examine the relationship between 24-hour urine volume and 28-day ICU mortality.

**Results:**

A negative correlation was found between 24-hour urine volume and ICU 28-day mortality. In the fully adjusted model, each 50 mL increase in 24-hour urine volume significantly reduced mortality risk by 1% (OR = 0.99, 95% CI = 0.98–0.99, *P* < 0.001). A nonlinear dose-response relationship was observed, with an inflection point at ~1,663.5 ml. Below this threshold, increased urine volume was significantly associated with reduced mortality risk (OR = 0.97, 95% CI: 0.96–0.98, *P* < 0.001), while above this point, the relationship was not statistically significant.

**Conclusion:**

This study demonstrates a non-linear negative correlation between 24-hour urine volume and 28-day ICU mortality in sepsis patients.

## Introduction

Sepsis is defined as a dysregulated host response to infection that results in life-threatening organ dysfunction (1). In the United States, the cost of hospitalization due to sepsis is significant, with expenses exceeding $23 billion (6.2%) in 2013 (2). Despite the considerable healthcare resources consumed, the mortality rate from sepsis has not significantly improved in recent years (3).

The literature indicates that the incidence of acute kidney injury (AKI) in sepsis patients ranges from 25 to 75% (4). According to the definition by the Acute Disease and Quality Initiative (ADQI) Group, Sepsis-associated acute kidney injury (SA-AKI) is a heterogeneous syndrome resulting from direct mechanisms related to infection or the host's response to infection, as well as indirect mechanisms caused by adverse effects of sepsis or its treatments (5, 6). SA-AKI has been shown to have a worse prognosis compared to patients with other conditions in the intensive care unit (ICU) (5). It is associated with prolonged ICU and hospital stays, higher mortality rates, increased hospitalization costs, greater long-term disability, and reduced quality of life across different populations (7, 8). Therefore, it is crucial to identify sepsis patients at risk of developing SA-AKI early and provide timely interventions to improve patient outcomes.

The 24-hour urine volume and serum creatinine levels are the most commonly used clinical indicators for assessing AKI and are also key factors in evaluating patient prognosis (7). However, in patients with sepsis, reduced urine volume has greater sensitivity and specificity than serum creatinine levels (9). This is because, in sepsis, decreased muscle perfusion leads to reduced creatine production, slowing the increase in serum creatinine levels and limiting its effectiveness in early AKI assessment (10).

However, to date, there have been few studies examining the relationship between 24-hour urine volume and mortality. Avila, Maria O. N., and colleagues (11) followed 879 patients with acute kidney injury and used logistic regression analysis to explore the association between 24-hour urine volume and mortality. The study found a negative correlation between urine volume and mortality risk. However, it is worth noting that due to the limitations of generalized linear models, potential non-linear relationships between variables may not be detected. Although various clinical scoring systems, such as the Quick Sequential Organ Failure Assessment (qSOFA) (12) and the New York Sepsis Severity Score (13), as well as serum biomarkers including procalcitonin, erythropoietin, and pentraxin-3, are effective for early prognosis assessment in sepsis patients (14), most primary care hospitals lack the capability to measure these biomarkers. In contrast, 24-hour urine volume is a common and widely applicable indicator. Investigating the relationship between 24-hour urine volume and mortality can help clinicians better understand its clinical significance. Therefore, this study aims to analyze the association between 24-hour urine volume and ICU mortality in sepsis patients using the eICU database and explore any potential dose-response relationship.

## Methods

### Data source

This study is a multicenter retrospective cohort analysis. The data were sourced from the eICU remote monitoring system developed by Philips Healthcare and established in collaboration with the eICU Research Institute (eRI) and The Laboratory for Computational Physiology at the Massachusetts Institute of Technology. The eICU Collaborative Research Database (eICU-CRD) comprises de-identified health data from over 200,000 ICU admissions of ~140,000 unique patients in the United States between 2014 and 2015 (15). The database includes vital signs measurements, care plan documents, disease severity assessments, and diagnostic and treatment information collected through the Philips eICU system. To ensure patient confidentiality and eliminate the need for additional informed consent, all patient information used in this study has been de-identified. The data were accessed and extracted in accordance with the data usage protocol of the PhysioNet Review Committee, following examination and certification (our record ID: 40859994). Access to this database was granted upon registration with eICU, thus no ethical review or patient consent documents from the author's institution were required.

### Patient selection

This study population comprised all patients admitted with a diagnosis of sepsis. Patients were identified using the International Classification of Diseases, Ninth Revision, Clinical Modification (ICD-9-CM) codes for sepsis, and only those with a primary diagnosis were included. Sepsis was diagnosed based on the presence of a suspected or confirmed infection along with a rapid increase of more than two points in the Sequential Organ Failure Assessment (SOFA) score, which is derived from the Acute Physiology and Chronic Health Evaluation (APACHE) IV scale (16, 17).

Exclusion criteria were as follows: (1) records of patients not admitted to the ICU for the first time; (2) patients with an ICU stay of <24 h; (3) patients younger than 18 years; (4) patients with indeterminate survival status; (5) patients lacking 24-hour urine volume data after ICU admission.

### Study variables

The eICU database encompasses a wide array of patient information, including basic demographic data, physiological monitoring parameters from bedside monitors, diseases diagnosed according to ICD-9-CM codes, and laboratory results collected during routine medical care.

We collected data from the first 24 h after ICU admission. Baseline characteristics, including age, gender, race, and body mass index (BMI). Vital signs data within 24 h of ICU admission included temperature, respiratory rate, heart rate, and mean arterial pressure (MAP). Pre-admission comorbidities included diabetes, liver failure, cirrhosis, and metastatic cancer. The treatment interventions within 24 h of ICU admission included intubation, mechanical ventilation, dialysis, and vasopressor use. The first laboratory measurements within 24 h after ICU admission, including serum albumin, serum creatinine, alanine aminotransferase (ALT), aspartate aminotransferase (AST), and total cholesterol (TC), were obtained from the relevant laboratory records. Upon ICU admission, a series of severity scores were recorded, including the SOFA score, APACHE IV score, Glasgow Coma Scale (GCS) score, and Acute Physiology Score III.

The exposure variable in this study, 24-hour urine volume, was the total amount of urine produced during the first 24 h after ICU admission. Based on the study by Avila et al. (11), we categorized the population into five groups: anuria (urine volume ≤ 50 mL/24 h), oliguria (urine volume >50 mL/24 h and <400 mL/24 h), and non-oliguric groups further divided into ≥400 mL/24 h and ≤ 1,000 mL/24 h, >1,000 mL/24 h and ≤ 2,000 mL/24 h (Group 4), and >2,000 mL/24 h (Group 5).

### Outcome

The outcome of this study was 28-day mortality after ICU admission.

### Statistical analysis

Continuous variables were described using the mean ± SD or median with interquartile range (IQR). Categorical variables were presented as counts and percentages. One-way analysis of variance was used to assess differences in normally distributed continuous variables among different urine volume groups. The Kruskal-Wallis test was used to assess differences in non-normally distributed continuous variables among these groups. Chi-square tests or Fisher's exact tests were employed to evaluate differences in categorical variables. A multivariate logistic regression model was employed to estimate the correlation between 24-hour urine volume and 28-day mortality. The results are expressed as OR with their 95% confidence intervals (95% CI). Following the STROBE guidelines, we presented the results of multivariate regression analyses, including unadjusted, minimally adjusted, and fully adjusted models (18). Covariate adjustment was guided by clinical significance, with adjustments made if the matched odds ratio (OR) changed by at least 10% (19).

A generalized additive model (GAM) with curve fitting was used to explore the dose-response relationship between 24-hour urine volume and 28-day mortality. Subsequently, a two-piecewise linear regression model was employed to investigate the threshold effect of 24-hour urine volume on mortality. When a clear non-linear relationship between 24-hour urine volume and 28-day mortality was observed on the smoothed curve, the software automatically calculated the inflection point using a recursive method after adjusting for potential confounders. Furthermore, a log-likelihood ratio test was conducted to compare the generalized linear model with the two-part linear model. The statistical analyses of this study were conducted using the R software, version 4.2.0 (R Foundation), and the EmpowerStats software (http://www.empowerstats.com, X&Y Solutions, Inc., Boston, MA). The level of statistical significance was set at *P* < 0.05.

## Results

### Baseline demographic and clinical characteristics

**Figure 1:**
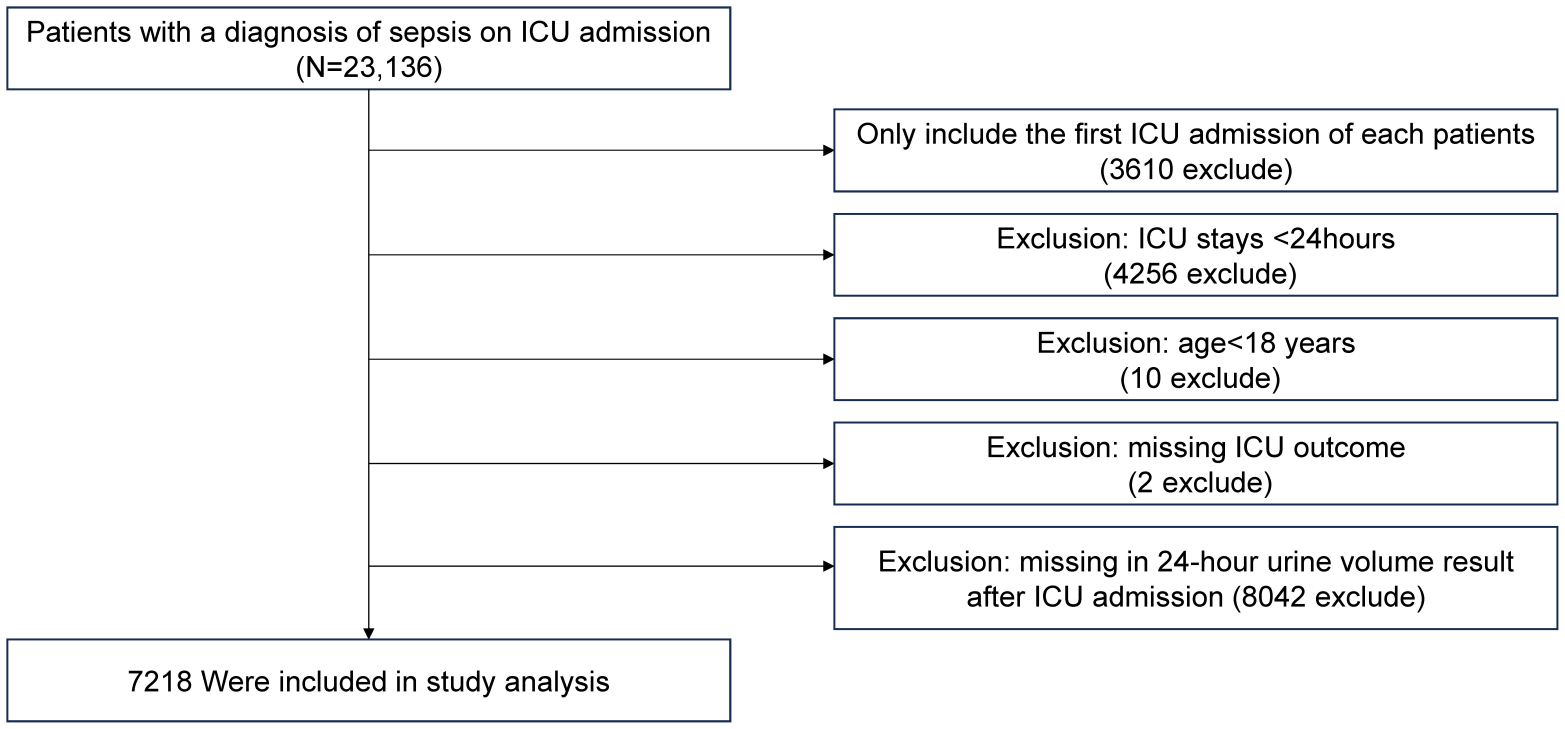
Flowchart of the study population.

The inclusion and exclusion criteria for the study population are illustrated in Figure 1. A total of 7,218 patients were included in the study, with baseline characteristics summarized in Table 1. The data show that patients with lower 24-hour urine volume tend to be older, have a lower proportion of females (*P* = 0.005), and have lower body temperature and mean arterial pressure (*P* < 0.001). These patients also had higher severity scores (APACHE IV, SOFA, Acute Physiology Score III), worse lab results such as kidney function (elevated BUN, creatinine, and anion gap), and electrolyte imbalances (potassium, chloride, sodium). The 28-day ICU mortality rate was 20.32% in the <50 ml/24 h urine group, compared to 3.82% in the highest urine volume group. Patients with lower urine volume required more interventions, including mechanical ventilation, intubation, and dialysis. Comorbidities such as liver failure, cirrhosis, acute myocardial infarction, and diabetes were more common in the low urine volume group. There were no statistically significant differences in ICU type among the groups (*P* = 0.068).

**Table 1:** Baseline demographic and clinical characteristics of patients.

	**Urine volume**					***P*-value**
**Variables**	<**50**	≥**50**,<**400**	≥**400**,<**1,000**	≥**1,000**,<**2,000**	≥**2,000**	
Number	379	863	1,649	2,136	2,119	
**Demographics** ^*^						
Age (years, mean ± SD)	65.48 ± 15.07	69.36 ± 14.53	68.75 ± 15.60	66.29 ± 15.89	60.65 ± 16.87	<0.001
Gender (*N*, %)						0.005
Male	189 (49.87)	446 (51.68)	784 (47.57)	997 (46.68)	940 (44.36)	
Female	190 (50.13)	417 (48.32)	864 (52.43)	1,139 (53.32)	1,179 (55.64)	
Ethnicity (*N*, %)						<0.001
Caucasian	256 (67.90)	667 (78.10)	1,273 (77.91)	1,646 (77.68)	1,584 (75.14)	
African American	67 (17.77)	103 (12.06)	170 (10.40)	200 (9.44)	209 (9.91)	
Hispanic	15 (3.98)	36 (4.22)	80 (4.90)	95 (4.48)	140 (6.64)	
Asian	30 (7.96)	26 (3.04)	82 (5.02)	127 (5.99)	115 (5.46)	
Native American	5 (1.33)	9 (1.05)	10 (0.61)	14 (0.66)	14 (0.66)	
Other/Unknown	4 (1.06)	13 (1.52)	19 (1.16)	37 (1.75)	46 (2.18)	
Admission weight (kg, mean ± SD)	82.13 ± 29.88	81.91 ± 28.07	79.40 ± 26.32	81.56 ± 26.57	82.91 ± 26.79	0.003
Admission height (cm, mean ± SD)	167.94 ± 11.84	168.04 ± 11.44	168.83 ± 11.30	168.96 ± 11.14	169.41 ± 11.37	0.017
BMI (mean ± SD)	29.10 ± 10.21	28.99 ± 9.45	27.75 ± 8.41	28.48 ± 8.69	28.83 ± 8.62	<0.001
**Vital signs** ^*^						
Temperature (°C, mean ± SD)	36.20 ± 1.17	36.42 ± 1.23	36.56 ± 1.18	36.63 ± 1.09	36.73 ± 1.13	<0.001
Respiratory rate (bpm, mean ± SD)	27.25 ± 14.08	31.34 ± 14.04	30.28 ± 14.11	30.37 ± 14.22	29.93 ± 14.35	<0.001
Heart rate (/min)	111.91 ± 28.56	113.59 ± 30.88	113.68 ± 29.11	112.33 ± 28.56	113.69 ± 26.94	0.407
MAP (mmHg, median Q1–Q3)	51.00 (43.50–65.00)	53.00 (45.00–100.25)	55.00 (47.00–73.00)	57.00 (48.00–108.50)	58.00 (49.00–112.00)	<0.001
**Severity of illness** ^*^						
GCS score (mean ± SD)	12.22 ± 3.46	11.96 ± 3.65	12.43 ± 3.46	12.87 ± 3.16	13.02 ± 3.06	<0.001
Acute Physiology Score III (mean ± SD)	78.82 ± 27.05	79.60 ± 25.71	62.55 ± 23.26	54.95 ± 21.26	47.99 ± 20.60	<0.001
SOFA score (mean ± SD)	7.39 ± 2.65	6.93 ± 2.86	4.46 ± 2.83	3.48 ± 2.49	3.28 ± 2.49	<0.001
Apache IV score (mean ± SD)	92.31 ± 27.83	94.83 ± 26.04	77.79 ± 24.55	68.81 ± 22.83	59.21 ± 22.24	<0.001
**Laboratory tests** ^*^						
Blood urea nitrogen (mg/dL, median Q1–Q3)	39.00 (23.00–57.00)	36.00 (23.00–56.00)	30.00 (18.00–47.00)	25.00 (16.00–41.00)	21.00 (13.00–36.00)	<0.001
**Variables**	<**50**	≥**50**,<**400**	≥**400**,<**1,000**	≥**1,000**,<**2,000**	≥**2,000**	
Serum creatinine (mg/dL, mean ± SD)	3.62 ± 2.71	2.50 ± 1.95	1.74 ± 1.40	1.47 ± 1.12	1.41 ± 1.23	<0.001
Bicarbonate (mmol/L, mean ± SD)	20.48 ± 5.77	20.85 ± 5.44	21.74 ± 5.52	22.45 ± 5.08	22.63 ± 5.27	<0.001
Glucose (mg/dl, median Q1–Q3)	122.00 (95.00–172.75)	129.00 (100.00–178.00)	125.00 (99.00–160.00)	125.00 (101.00–167.00)	125.00 (101.00–166.00)	0.572
Serum potassium (mmol/L, mean ± SD)	4.35 ± 0.91	4.28 ± 0.85	4.09 ± 0.75	4.01 ± 0.70	3.97 ± 0.73	<0.001
Chloride (mmol/L, mean ± SD)	103.06 ± 7.82	104.48 ± 7.84	105.77 ± 7.98	105.87 ± 7.18	105.74 ± 7.44	<0.001
Calcium (mg/dl, mean ± SD)	8.02 ± 0.90	7.91 ± 0.88	7.95 ± 0.89	7.93 ± 0.82	7.92 ± 0.79	0.242
Sodium (mmol/L, mean ± SD)	137.16 ± 6.02	137.59 ± 6.78	138.45 ± 6.93	138.27 ± 6.15	138.15 ± 6.05	<0.001
Anion gap (mmol/L, mean ± SD)	14.04 ± 6.45	13.03 ± 5.09	11.55 ± 4.82	10.71 ± 4.11	10.84 ± 4.50	<0.001
Total protein (g/dL, mean ± SD)	5.65 ± 1.00	5.57 ± 1.02	5.60 ± 0.87	5.64 ± 0.87	5.76 ± 0.86	<0.001
Albumin (g/dL, mean ± SD)	2.48 ± 0.72	2.47 ± 0.60	2.46 ± 0.61	2.52 ± 0.59	2.59 ± 0.59	<0.001
Hct (%, mean ± SD)	30.93 ± 6.43	31.68 ± 6.39	31.72 ± 6.46	31.94 ± 6.36	31.62 ± 6.22	0.026
Urine volume (ml, mean ± SD)	11.05 ± 15.62	227.87 ± 101.76	701.92 ± 171.25	1,439.54 ± 282.13	3,258.76 ± 1,113.12	<0.001
**Clinical data** ^*^						
Source of infection (*N*, %)						<0.001
Pulmonary	89 (23.48)	306 (35.46)	616 (37.36)	844 (39.51)	770 (36.34)	
Renal/UTI (including bladder)	64 (16.89)	206 (23.87)	382 (23.17)	472 (22.10)	514 (24.26)	
Gastrointestinal	62 (16.36)	114 (13.21)	234 (14.19)	255 (11.94)	199 (9.39)	
Cutaneous/soft tissue	35 (9.23)	53 (6.14)	116 (7.03)	160 (7.49)	194 (9.16)	
Gynecologic	0 (0.00)	3 (0.35)	4 (0.24)	12 (0.56)	8 (0.38)	
Other/unknown	129 (34.04)	181 (20.97)	297 (18.01)	393 (18.40)	434 (20.48)	
Unit type (*N*, %)						0.068
Medical-surgical ICU	247 (65.17)	596 (69.06)	1,098 (66.59)	1,429 (66.90)	1,386 (65.41)	
Neurological ICU	45 (11.87)	113 (13.09)	220 (13.34)	266 (12.45)	262 (12.36)	
Coronary care unit/cardiothoracic ICU	29 (7.65)	44 (5.10)	91 (5.52)	107 (5.01)	150 (7.08)	
Cardiothoracic ICU	33 (8.71)	57 (6.60)	125 (7.58)	173 (8.10)	174 (8.21)	
Medical ICU	15 (3.96)	28 (3.24)	66 (4.00)	95 (4.45)	99 (4.67)	
Surgical ICU	7 (1.85)	7 (0.81)	31 (1.88)	25 (1.17)	25 (1.18)	
Cardiac surgery ICU	2 (0.53)	8 (0.93)	5 (0.30)	18 (0.84)	7 (0.33)	
**Variables**	<**50**	≥**50**,<**400**	≥**400**,<**1,000**	≥**1,000**,<**2,000**	≥**2,000**	
Cardiac ICU	1 (0.26)	10 (1.16)	13 (0.79)	23 (1.08)	16 (0.76)	
**Treatment data** ^*^						
Intubated (*N*, %)						<0.001
No	299 (78.89)	671 (77.75)	1,345 (81.56)	1,781 (83.38)	1,837 (86.69)	
Yes	80 (21.11)	192 (22.25)	304 (18.44)	355 (16.62)	282 (13.31)	
Mechanical ventilation use (*N*, %)						<0.001
No	265 (69.92)	599 (69.41)	1,209 (73.32)	1,606 (75.19)	1,655 (78.10)	
Yes	114 (30.08)	264 (30.59)	440 (26.68)	530 (24.81)	464 (21.90)	
Dialysis (*N*, %)						<0.001
No	290 (76.52)	798 (92.47)	1,621 (98.30)	2,126 (99.53)	2,113 (99.72)	
Yes	89 (23.48)	65 (7.53)	28 (1.70)	10 (0.47)	6 (0.28)	
Vasopressor use (*N*, %)						0.907
No	374 (98.68)	842 (98.59)	1,619 (98.60)	2,102 (98.64)	2,093 (98.91)	
Yes	5 (1.32)	12 (1.41)	23 (1.40)	29 (1.36)	23 (1.09)	
**Comorbidities** ^*^						
Hepatic failure (*N*, %)						<0.001
No	369 (97.36)	834 (96.64)	1,601 (97.09)	2,099 (98.27)	2,102 (99.20)	
Yes	10 (2.64)	29 (3.36)	48 (2.91)	37 (1.73)	17 (0.80)	
Metastatic cancer (*N*, %)						0.902
No	365 (96.31)	835 (96.76)	1,593 (96.60)	2,066 (96.72)	2,057 (97.07)	
Yes	14 (3.69)	28 (3.24)	56 (3.40)	70 (3.28)	62 (2.93)	
Cirrhosis (*N*, %)						<0.001
No	365 (96.31)	831 (96.29)	1,584 (96.06)	2,089 (97.80)	2,099 (99.06)	
Yes	14 (3.69)	32 (3.71)	65 (3.94)	47 (2.20)	20 (0.94)	
Diabetes (*N*, %)						<0.001
No	299 (78.89)	724 (83.89)	1,439 (87.27)	1,853 (86.75)	1,820 (85.89)	
Yes	80 (21.11)	139 (16.11)	210 (12.73)	283 (13.25)	299 (14.11)	
Congestive heart failure (*N*, %)						0.088
**Variables**	<**50**	≥**50**,<**400**	≥**400**,<**1,000**	≥**1,000**,<**2,000**	≥**2,000**	
No	335 (88.39)	801 (92.82)	1,503 (91.15)	1,945 (91.06)	1,950 (92.02)	
Yes	44 (11.61)	62 (7.18)	146 (8.85)	191 (8.94)	169 (7.98)	
Acute myocardial infarction (*N*, %)						0.031
No	373 (98.42)	830 (96.18)	1,598 (96.91)	2,054 (96.16)	2,066 (97.50)	
Yes	6 (1.58)	33 (3.82)	51 (3.09)	82 (3.84)	53 (2.50)	
**Outcome**						
ICU 28 day mortality (*N*, %)						<0.001
No	302 (79.68)	692 (80.19)	1,464 (88.78)	2,010 (94.10)	2,038 (96.18)	
Yes	77 (20.32)	171 (19.81)	185 (11.22)	126 (5.90)	81 (3.82)	

MAP, mean arterial pressure; BMI, body mass index; GCS, Glasgow coma scale; SOFA, Sequential Organ Failure Assessment; Hct, hematocrit; UTI, urinary tract infection.

^*^Data were collected within the first 24 h of ICU admission.

### Univariate regression analysis of baseline variables and 28-day ICU mortality

The results of the univariate regression analysis are presented in Table 2. Increased age, elevated respiratory rate, elevated heart rate, higher disease severity scores (APACHE IV, SOFA, Acute Physiology Score III), intubation, mechanical ventilation, use of vasopressors, elevated blood urea nitrogen, serum creatinine, serum potassium, and anion gap were all positively correlated with increased mortality (all *P* < 0.001). Additionally, comorbidities at admission, such as liver failure, metastatic cancer, cirrhosis, congestive heart failure, and acute myocardial infarction, were also positively associated with mortality risk (all *P* < 0.05). In contrast, body temperature, GCS score, urine volume, calcium, bicarbonate, total protein, albumin levels, and a history of diabetes were negatively correlated with mortality (all *P* < 0.001). Compared to pulmonary infections, mortality was lower in patients with renal/urinary tract infections and gynecological infections (*P* < 0.001).

**Table 2:** Univariate regression analysis of baseline variables and 28-day mortality.

	**Statistics**	**OR (95% CI) *P***
Age	65.43 ± 16.31	1.01 (1.01, 1.02) <0.001
**Gender**		
Male	3,380 (46.83%)	1.0
Female	3,837 (53.17%)	0.95 (0.81, 1.11) 0.515
**Ethnicity**		
Caucasian	5,478 (76.47%)	1.0
African American	756 (10.55%)	0.95 (0.72, 1.24) 0.698
Hispanic	374 (5.22%)	0.83 (0.56, 1.23) 0.359
Asian	385 (5.37%)	0.75 (0.50, 1.12) 0.156
Native American	52 (0.73%)	0.61 (0.19, 1.95) 0.403
Other/unknown	119 (1.66%)	0.71 (0.35, 1.47) 0.362
Admission weight	81.57 ± 26.97	1.00 (0.99, 1.00) 0.023
Admission height	168.92 ± 11.32	1.00 (0.99, 1.00) 0.549
BMI	28.51 ± 8.80	0.99 (0.98, 1.00) 0.085
Temperature	36.60 ± 1.15	0.82 (0.76, 0.88) <0.001
Respiratory rate	30.19 ± 14.21	1.02 (1.01, 1.02) <0.001
Heart rate	113.19 ± 28.46	1.01 (1.01, 1.02) <0.001
MAP	75.98 ± 42.28	1.00 (1.00, 1.00) 0.017
**Unit type**		
Medical-surgical ICU	4,804 (66.56%)	1.0
Neurological ICU	913 (12.65%)	1.21 (0.96, 1.53) 0.108
Coronary care unit/cardiothoracic ICU	427 (5.92%)	0.82 (0.56, 1.19) 0.296
Cardiothoracic ICU	568 (7.87%)	1.05 (0.78, 1.42) 0.743
Medical ICU	305 (4.23%)	1.01 (0.67, 1.52) 0.948
Surgical ICU	98 (1.36%)	0.93 (0.45, 1.93) 0.841
Cardiac surgery ICU	40 (0.55%)	1.84 (0.77, 4.41) 0.171
Cardiac ICU	63 (0.87%)	0.71 (0.26, 1.96) 0.505
**Source of infection**		
Pulmonary	2,646 (36.66%)	1.0
Renal/UTI (including bladder)	1,662 (23.03%)	0.39 (0.30, 0.51) <0.001
Gastrointestinal	868 (12.03%)	0.88 (0.69, 1.14) 0.337
Cutaneous/soft tissue	1,451 (20.10%)	0.96 (0.78, 1.18) 0.701
Gynecologic	564 (7.81%)	0.45 (0.31, 0.66) <0.001
Other/Unknown	27 (0.37%)	0.64 (0.15, 2.73) 0.549
**GCS score**		
Low	2,181 (30.71%)	1.0
Middle	1,579 (22.23%)	0.53 (0.42, 0.65) <0.001
High	3,342 (47.06%)	0.35 (0.29, 0.42) <0.001
**Acute physiology score III**		
Low	2,110 (31.92%)	1.0
Middle	2,293 (34.68%)	2.09 (1.50, 2.90) <0.001
High	2,208 (33.40%)	9.09 (6.78, 12.18) <0.001
**SOFA score**		
Low	1,565 (21.68%)	1.0
Middle	2,599 (36.01%)	1.81 (1.29, 2.54) 0.006
High	3,054 (42.31%)	5.89 (4.32, 8.02) <0.001
**Apache IV score**		
Low	2,185 (33.05%)	1.0
Middle	2,201 (33.29%)	2.12 (1.53, 2.93) <0.001
High	2,225 (33.66%)	8.77 (6.59, 11.67) <0.001
**Intubated**		
No	6,001 (83.14%)	1.0
Yes	1,217 (16.86%)	3.60 (3.03, 4.28) <0.001
**Mechanical ventilation use**		
No	5,392 (74.70%)	1.0
Yes	1,826 (25.30%)	2.89 (2.45, 3.41) <0.001
**Dialysis**		
No	7,020 (97.26%)	1.0
Yes	198 (2.74%)	0.71 (0.40, 1.26) 0.243
**Vasopressor use**		
No	7,102 (98.72%)	1.0
Yes	92 (1.28%)	2.20 (1.27, 3.79) 0.005
Blood urea nitrogen	33.65 ± 25.35	1.01 (1.01, 1.02) <0.001
Serum creatinine	1.75 ± 1.56	1.16 (1.11, 1.21) <0.001
Glucose	147.47 ± 86.99	1.00 (1.00, 1.00) 0.828
Total protein	5.66 ± 0.90	0.59 (0.52, 0.66) <0.001
Albumin	2.52 ± 0.60	0.54 (0.46, 0.63) <0.001
Sodium	138.13 ± 6.38	1.01 (1.00, 1.02) 0.090
Serum potassium	4.07 ± 0.76	1.37 (1.24, 1.51) <0.001
Chloride	105.48 ± 7.60	1.00 (0.99, 1.01) 0.934
Calcium	7.94 ± 0.85	0.81 (0.73, 0.90) <0.001
Anion gap	11.36 ± 4.73	1.08 (1.06, 1.10) <0.001
Hct	31.70 ± 6.34	1.00 (0.99, 1.01) 0.786
Bicarbonate	22.04 ± 5.38	0.93 (0.92, 0.95) <0.001
Urine volume/50 ml	31.73 ± 26.61	0.97 (0.96, 0.97) <0.001
**Hepatic failure**		
No	7,076 (98.03%)	1.0
Yes	142 (1.97%)	2.12 (1.36, 3.32) 0.001
**Metastatic cancer**		
No	6,986 (96.79%)	1.0
Yes	232 (3.21%)	1.54 (1.04, 2.29) 0.030
**Cirrhosis**		
No	7,040 (97.53%)	1.0
Yes	178 (2.47%)	1.78 (1.17, 2.72) 0.008
**Congestive heart failure**		
No	6,601 (91.45%)	1.0
Yes	617 (8.55%)	1.52 (1.18, 1.96) <0.001
**Acute myocardial infarction**		
No	6,993 (96.88%)	1.0
Yes	225 (3.12%)	1.93 (1.34, 2.80) <0.001
**Diabetes**		
No	6,200 (85.90%)	1.0
Yes	1,018 (14.10%)	0.73 (0.56, 0.94) 0.014

MAP, mean arterial pressure; BMI, body mass index; GCS, Glasgow coma scale; SOFA, Sequential Organ Failure Assessment; Hct, hematocrit; UTI, urinary tract infection.

### Results of 24-hour urine volume and ICU hospitalization death in different regression models

The relationship between 24-hour urine volume and ICU mortality across different adjustment strategies in the logistic regression models is presented in Table 3. The regression analysis results indicate that urine volume is negatively associated with mortality risk across all models: unadjusted, minimally adjusted, and fully adjusted. In the fully adjusted model (adjusted for age; ethnicity; BMI; unit type; Apache IV score; temperature; respiratory rate; heart rate; MAP; CHF; COPD; AMI; DM; calcium; bicarbonate; glucose; serum creatinine; anion gap; Vasopressor use), each 50 mL increase in 24-hour urine volume significantly reduced mortality risk by 1% (OR = 0.99, 95% CI = 0.98–0.99, *P* < 0.001). Compared to the reference group with normal urine volume (1,000–2,000 mL), the group with urine volume <50 mL had the highest mortality risk (OR = 2.63, 95% CI: 1.62–4.29, *P* < 0.001), while the group with urine volume ≥2,000 mL had a lower mortality risk (OR = 0.84, 95% CI: 0.56–1.26). A significant trend was observed in the fully adjusted model (*P* < 0.001).

**Table 3:** 24-hour urine volume and 28-day ICU death in different logistic regression models.

**Exposure**	**Non-adjusted, OR (95% CI) *P***	**Adjust I, OR (95% CI) *P***	**Adjust II, OR (95% CI) *P***
Urine volume/50 ml	0.97 (0.96, 0.97) <0.001	0.97 (0.96, 0.97) <0.001	0.99 (0.98, 0.99) <0.001
**Urine volume**			
≥1,000, <2,000	1.0	1.0	1.0
<50	4.07 (2.99, 5.54) <0.001	4.26 (3.12, 5.82) <0.001	2.63 (1.62, 4.29) <0.001
≥50, <400	3.94 (3.08, 5.04) <0.001	3.90 (3.04, 5.01) <0.001	1.86 (1.28, 2.69) 0.001
≥400, <1,000	2.02 (1.59, 2.55) <0.001	2.03 (1.60, 2.57) <0.001	1.59 (1.14, 2.22) 0.006
≥2,000	0.63 (0.48, 0.84) 0.002	0.66 (0.50, 0.89) 0.006	0.84 (0.56, 1.26) 0.392
*P* for trend	<0.001	<0.001	<0.001

Non-adjusted model adjust for: None.

Adjust I model adjust for: gender; age; ethnicity.

Adjust II model adjust for: gender; age; ethnicity; BMI; unit type; Apache IV score; temperature; respiratory rate; heart rate; MAP; CHF; COPD; AMI; DM; calcium; bicarbonate; glucose; serum creatinine; anion gap; Vasopressor use.

### The dose-response relationship between 24-hour urine volume and 28-day mortality

**Figure 2:**
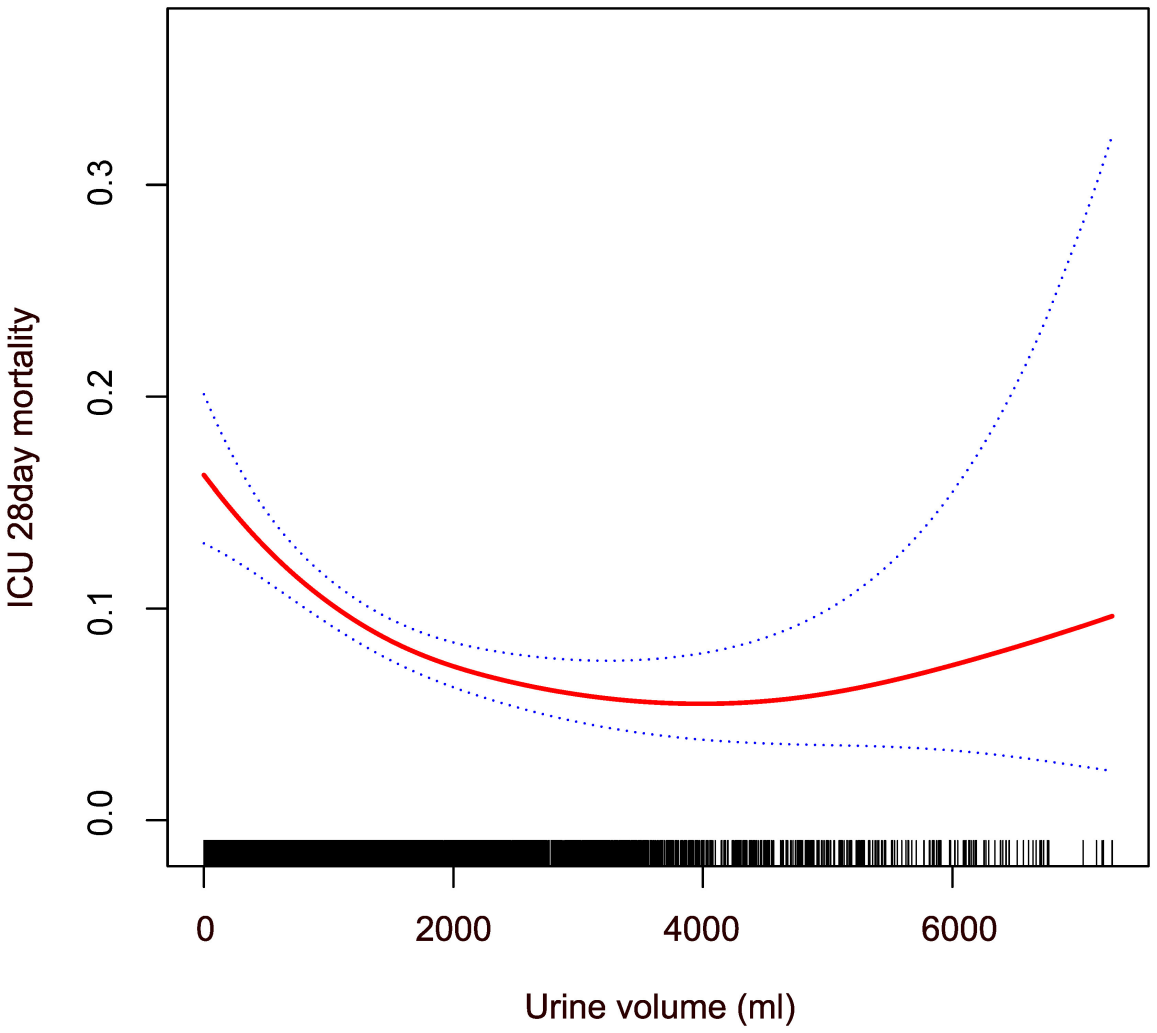
Non-linear relationship between 24-hour urine volume and 28-day mortality in patients with sepsis in ICU. The red line shows the smooth curve between the variables. The blue line represents the 95% confidence interval of the fit. Adjusting gender; age; ethnicity; BMI; unit type; Apache IV score; temperature; respiratory rate; heart rate; MAP; CHF; COPD; AMI; DM; calcium; bicarbonate; glucose; serum creatinine; anion gap; vasopressor use.

Using a fully adjusted model, we applied GAM and curve fitting to examine the non-linear relationship between 24-hour urine volume upon ICU admission and 28-day mortality in the ICU (Figure 2). As shown in the figure, the 28-day mortality rate in the ICU decreased with increasing 24-hour urine volume. However, this negative relationship weakened after reaching a certain level. We then used a two-piecewise linear regression model to analyze the potential dose-response relationship between the two variables (Table 4). In Model II, the calculated inflection point was 1,663.5 (33.27^*^50). On the left side of this inflection point, there was a significant association between 24-hour urine volume and reduced mortality risk (OR = 0.97, 95% CI 0.96–0.98, *P* < 0.001). On the right side of the inflection point, the relationship between the two variables was not statistically significant (OR = 1.01, 95% CI 0.98–1.04, *P* = 0.680).

**Table 4:** Results of a two-piece-wise linear regression model.

	**OR (95% CI)**	***P***
**Model 1**		
	0.98 (0.97, 0.99)	<0.001
**Model 2**		
Inflection point (K)	33.27	
<K	0.97 (0.96, 0.98)	<0.001
>K	1.01 (0.98, 1.04)	0.680
Log-likelihood ratio test		0.070

Exposure: 24 h urine volume/50 ml.

Outcome: ICU 28 mortality.

Model 1 was fitted using standard linear regression.

Model 2 was fitted using two-piecewise linear regression.

Both model 1 and model 2 was adjusted for: gender; age; ethnicity; BMI; unit type; Apache IV score; temperature; respiratory rate; heart rate; MAP; CHF; COPD; AMI; DM; calcium; bicarbonate; glucose; serum creatinine; anion gap; vasopressor use.

## Discussion

One advantage of this study is the use of a multicenter cohort from a publicly available database, which includes data from 7,218 ICU sepsis patients. Our findings indicate a negative correlation between urine output in the first 24 h after ICU admission and the 28-day mortality rate, with a non-linear relationship observed between the two. Patients with low urine volume typically exhibit more severe clinical characteristics, including older age, lower body temperature and mean arterial pressure, and higher disease severity scores (APACHE IV, SOFA, and Acute Physiology Score III). The GAM revealed a non-linear relationship between urine volume and mortality. Using a piecewise linear regression model, we found that before reaching an approximate urine volume of 1,663.5 ml (33.27^*^50 ml), increased urine volume was significantly associated with reduced mortality risk. However, beyond this threshold, the relationship was no longer significant.

To date, there is limited research on the correlation between urine volume and mortality. A study by Avila et al. (11) found a significant negative correlation between urine volume and mortality in patients with acute kidney injury, which is consistent with our findings. Additionally, in their study, multivariate regression results show that patients with anuria and oliguria have increased mortality risk by 95% (OR = 1.95, 95% CI: 1.06–3.60, *P* = 0.032) and 76% (OR = 1.76, 95% CI: 1.02–3.03, *P* = 0.042), respectively. Patients with urine volume >1,000 mL/24 h have a 50–70% reduced mortality risk, and every 100 mL increase in urine volume is associated with a 7.2% decrease in mortality risk (*P* < 0.001). Hakemi et al. (20) investigated the relationship between baseline urine volume and mortality in 1,472 outpatient peritoneal dialysis patients. Using a Cox proportional hazards regression model, this study also found that patients with a baseline urine volume ≥1,000 mL/day had higher survival rates compared to those with <250 mL/day. Shafi et al. (21) conducted a multicenter cohort study including 734 hemodialysis patients from 81 clinics, examining the relationship between residual kidney function and mortality. This study also used a Cox proportional hazards regression model and found that hemodialysis patients with urine volume (defined as the ability to produce at least 250 mL of urine per day) had a 30% lower risk of all-cause mortality (HR = 0.70, 95% CI: 0.52–0.93). The results of these studies confirm that reduced urine volume is a risk factor for poor outcomes. The advantage of our study is that it extends these findings by identifying a non-linear (L-shaped curve) relationship between 24-hour urine volume and ICU mortality in septic patients, with an inflection point at 1,663.5. Our findings can help clinicians better understand the relationship between 24-hour urine volume and mortality in septic patients, based on existing theoretical knowledge.

Currently, there are two known mechanisms leading to poor prognosis in septic patients: inflammation (22) and microcirculatory dysfunction (23). In septic patients, dysregulation of the inflammatory response leads to organ dysfunction and poor prognosis. Inflammatory mediators are released into the vascular lumen, where they bind to membrane receptors on immune cells, initiating downstream signaling cascades that stimulate the synthesis and release of pro-inflammatory factors, exacerbating the patient's condition. Similar membrane-bound receptors are present in renal tubular epithelial cells (TECs), primarily TLR2, and TLR4. When inflammatory mediators pass through the glomeruli or adjacent peritubular capillaries, proximal TECs also exhibit increased oxidative stress, reactive oxygen species production, and mitochondrial damage (24, 25). Additionally, microthrombi formation and capillary blockage caused by endothelial damage, autonomic nervous system responses, glycocalyx shedding, and activation of the coagulation cascade lead to microcirculatory dysfunction, prolonging TEC exposure to activated circulating inflammatory mediators (24, 26). TECs can also inactivate adjacent cells by initiating paracrine signaling (27), and there is increased infiltration of inflammatory cells in the glomerular and peritubular regions (28). These factors lead to progressive deterioration of renal function and consequently reduced urine volume.

It is well-known that the volume of urine produced by the kidneys is closely related to the patient's systemic circulation, metabolism, and prognosis (29). Research has shown that in hemodialysis patients, those with residual urinary function can effectively avoid fluid overload and its complications, including left ventricular hypertrophy and uncontrolled hypertension (30). Considering the variability in baseline kidney function, we used serum creatinine levels at ICU admission as a proxy for baseline kidney function and adjusted for this variable in the multivariate regression model to account for differences in kidney function. Additionally, studies have demonstrated that vasopressor medications can significantly improve survival rates in septic patients and reduce the likelihood of renal replacement therapy (31, 32). Therefore, we also adjusted for the use of vasopressors as a covariate in the regression analysis.

This study has several limitations. First, our study focuses on the association between urine volume during the first 24 h of ICU admission and 28-day ICU mortality in septic patients. However, the evidence provided by urine volume on the 1st day is still limited. More data are needed to supplement the assessment of how changes in urine volume relate to disease prognosis. For example, considering urine volume on the 2nd day of admission or the relationship between urine volume changes and 28-day mortality would be beneficial. Additionally, due to limitations of the database, we could only extract the ICU 28-day survival outcomes for septic patients. Therefore, our conclusions are applicable only within this time frame, and further research is needed to extend the applicability of these findings. Furthermore, as an observational study, this research is subject to baseline confounding factors. Therefore, we adjusted for various confounding variables to ensure the consistency of the results. For instance, the use of vasopressors may impact disease prognosis, so we included their use as a covariate and found consistent results before and after adjustment. This indicates that after controlling for this confounding factor, the core findings of our study remain unchanged. Our study population consists of patients diagnosed with sepsis upon ICU admission, which limits the generalizability of our findings to this specific group. Future research should explore the relationship between 24-hour urine volume and 28-day ICU mortality in different populations to expand the evidence base.

## Conclusion

This study found a negative correlation between 24-hour urine volume and 28-day mortality in septic patients, with a non-linear dose-response relationship.

## Data Availability

The datasets presented in this study can be found in online repositories. The names of the repository/repositories and accession number(s) can be found at: https://physionet.org/content/eicu-crd/2.0/.
